# Assessment of Awareness Levels About Anticoagulants in Patients With Atrial Fibrillation Presenting to Emergency Department

**DOI:** 10.7759/cureus.12963

**Published:** 2021-01-28

**Authors:** Canan Akman, Bahadır Kırılmaz, Serdal Balcı, Ercan Aksit, Ersan Yurtseven, Ali Duygu

**Affiliations:** 1 Emergency Department, Canakkale Onsekiz Mart University Faculty of Medicine, Canakkale, TUR; 2 Cardiology Department, Canakkale Onsekiz Mart University Faculty of Medicine, Canakkale, TUR; 3 Emergency Department, Kocaeli Farabi Training and Research Hospital, Kocaeli, TUR

**Keywords:** atrial fibrillation, emergency department, anticoagulants

## Abstract

Atrial fibrillation (AF) is a rhythm disorder observed mostly amongst adults. AF has been regarded as one of the most important medical problems because it leads to thromboembolism and paralysis risks. Although warfarin has been used in the past to cope with this health problem, new oral anticoagulant medicines have replaced it in the last few years. The new oral anticoagulants, namely, dabigatran etexilate, rivaroxaban, and apixaban, are currently being used in daily clinical practice and treatment guidelines. Since AF patients are supposed to receive long-term oral anticoagulant therapy, it is extremely important to provide them with accurate information and appropriate training regarding the treatment to decrease oncoming complications.

This is a prospective study involving 168 patients who were admitted to the emergency department with AF and who were using oral anticoagulants. Findings indicate a lack of awareness in the patients regarding the effects and side effects of the drugs they take despite having been informed by the prescribing physician. We believe that informed action by patients with regard to the oral anticoagulants and their side effects will have an impact on the reduction in hospitalization observed. It will also make a substantial contribution to the quality of life of AF patients and to their use of medical services.

## Introduction

Atrial fibrillation (AF) is a rhythm disorder observed mostly among adults. Also referred to as supraventricular arrhythmia, AF is an important medical problem, as it may result in stroke and thromboembolism. Warfarin, which has been used in the treatment of atrial fibrillation in the past, is being replaced by a new generation of oral anticoagulants [1]. The clinical use of warfarin is plagued by difficulties and restrictions because it requires dose adjustments, routine monitoring of patients’ international normalized ratio (INR) measurements, and is dependent on food-drug interactions. For this reason, a new generation of oral anticoagulants without any drug interactions has been developed that can be easily managed up in the follow-up of patients. Research has led to a new generation of oral anticoagulants, such as dabigatran etexilate (Pradaxa), rivaroxaban (Xarelto), and apixaban (Eliquis), which are currently used in daily clinical practice and treatment guidelines. While these drugs are as effective as warfarin, they don't need additional dosage monitoring and have minimal food-drug interactions [2].

Guidelines recommend initiating warfarin or the new-generation oral anticoagulants in patients with atrial fibrillation who have a stroke risk. The common recommendation is to use the CHA2DS2-VASc (congestive heart failure, hypertension, age ≥ 75 years, diabetes mellitus, stroke or transient ischemic attack [TIA], vascular disease, age 65 to 74 years, sex category) scoring system to determine the risk of stroke and to initiate antithrombolytic therapy when drug therapy is initiated [3]. The CHA2DS2-VASc score is a validated tool to predict the risk of stroke and systemic emboli in patients with non-valvular atrial fibrillation. Since patients with AF will have to receive oral anticoagulant medication for the rest of their lives, it is important that they are informed about warfarin and other new-generation oral anticoagulant therapy. It is also important that their skills regarding the same are increased. In this way, there is a significant decrease in the complications caused by the drugs [4]. The aim of our study is to examine the awareness about anticoagulant drugs taken in patients with atrial fibrillation admitted to the emergency room of a university hospital.

## Materials and methods

This is a prospective study of 168 patients with a diagnosis of atrial fibrillation, taking oral anticoagulants, who were admitted to a university hospital in the Çanakkale province. After obtaining the approval of the ethics committee, a questionnaire was administered to the patients in the emergency department by an emergency medicine specialist. The participants were interviewed face to face after giving them information about the purpose and content of the study. The questionnaire included 28 questions, including the duration of oral anticoagulant medication taken, the side effects and interactions they experienced, and demographic data. Data were analysed using Statistical Package for the Social Sciences software, version 19.0 (IBM Corp., Armonk, New York, USA). Numbers, percentages, averages, standard deviations, minimums, and maximums have been used to present the metadata. Results were considered statistically significant if p<0.05.

## Results

The study included 168 patients, 88 (52%) women, and 80 (47.6%) men. The average age was 70.1±10.3 years (min 38, max 89). Of the study group, 116 (69%) had completed primary school, 82 (48.8%) were housewives, 101 (60.1%) were non-smokers, and 126 (75.0%) had never tried alcohol before (Table 1).

**Table 1:** Demographic data

Variable		n (%)
Education	Illiterate	27 (16.1)
	Primary School	116 (69.0)
	High School	19 (19.3)
	University	6 (3.6)
Occupation	Housewife	82 (48.8)
	Retiree	70 (41.7)
	Employee	16 9.5)
Smoking	Smokers	15 (8.9)
	Non-Smokers	101(60.1)
	Ex-smokers	52 (31)
Drinking	Drinker	10 (6.0)
	Non-Drinker	126 (75.0)
	Ex-Drinker	32 (19)

Of the study group, 38 (22.6%) took 5 mg warfarin and 31 (18.5%) took 2 mg rivoraksaban. Of all patients, 43 (25.6%) had received anticoagulant treatment for 6-12 months, 26 (15.5%) for 1-6 months, and seven (4.2%) for one week to one month (Table 2).

**Table 2:** Distribution of anticoagulant drugs

Variables	n (%)
Warfarin 5 mg	38 (22.6)
Rivoraxaban 20 mg	31 (18.5)
Dabigatran 150 mg	22 (13.1)
Rivoraxaban 15 mg	22 (13.1)
Apixaban 5 mg	21 (12.5)
Dabigatran 110 mg	19 (11.3)
Apixaban 2.5 mg	15 (8.9)
Duration of anticoagulant use	
1 week–1 month	7 (4.2)
1–6 months	26 (15.5)

Of all doctors who provided information about anticoagulant treatment, 131 (78%) were prescribing doctors, 18 (10.7%) were the doctor at the time of hospital admission, 18 (10.7%) were the doctor during outpatient control, and one (0.6%) was a family doctor. Of all patients, 114 (49.5%) suffered from hypertension, 37 (16.0%) suffered from diabetes mellitus, and 36 (15.6%) suffered from heart failure. Of all patients, 152 (90.5%) were receiving drugs in addition to anticoagulant treatment. Overall, 139 (82.7%) patients had no knowledge of the side effects of other drugs when taken with anticoagulants. Of all patients, 142 (82.0%) observed no side effects while receiving anticoagulant treatment, while eight (4.6%) reported excessive gingival and nasal bleeding, and eight (4.6%) observed large bruises and bumps on their bodies in the absence of any impact (Table 3).

**Table 3:** Side effects observed during anticoagulant treatment

Variables	n (%)
No side effects	142 (82.0)
Excessive gingival and nasal bleeding	8 (4.6)
Large bruises and bumps in the absence of any impact on the body	8 (4.6)
Vomiting and oral bleeding, red in colour and in the form of coffee grounds	4 (2.3)
Fecal bleeding or tar-like bleeding	4 (2.3)
Cough and oral bleeding in the form of a pinkish foam	3 (1.8)
Excessive long-lasting genital bleeding with plenty of clots	2 (1.2)
Hematuria	1 (0.6)
Allergic reactions	1 (0.6)

Ninety-nine patients (57.6%) forgot what effects they may encounter if the drug dose is forgotten, while 114 (67.9%) patients knew nothing about the consequences of excessive doses. Of patients in the study, 153 (91.1%) had no idea about the effects of other drugs on bleeding when taken with anticoagulant drugs, 159 (94.6%) knew nothing about how long it takes for the drug to be effective after being taken orally, and 158 (94.0%) had no idea when the duration of action would end after the drug was stopped. Of the patients, 28 (16.1%) stated that anticoagulant treatment is necessary for vascular diseases, 2 (1.1%) for heart failure, 10 (5.7%) for prosthetic heart valves, and 68 (39.2%) for cardiac rhythm disturbances. In total, 66 (37.9%) patients stated that they had no diseases that required anticoagulant therapy. 92.7% (n = 114) of patients using warfarin or using warfarin earlier were regularly monitored for INR. Of patients whose INR was regularly monitored, 95 (77.2%) had their levels checked once a month, while the remaining 12 (9.8%) had theirs checked every week. Of the patients using warfarin, 27 (71.1%) had to inform the doctor the name of the drug whenever they sought help at a healthcare organization. With regard to when warfarin dose should be adjusted, 35.9% (n = 46) of the patients said before any surgical intervention, 17.2% (n = 22) said during tooth extraction, 1.6% (n = 2) said in case of bleeding, and 45.3% (n = 58) did not know under which circumstances dose adjustment should be made. In total, 97 (78.8%) patients reported that the cardiologist who initiated the treatment should decide on dosage changes.

## Discussion

Atrial fibrillation (AF) is a completely irregular, variable arrhythmia with low amplitude on the electrocardiogram (ECG) without effective atrial contraction [5]. AF is the most common cardiac arrhythmia, and its prevalence increases with age. While the prevalence in under 55 years of age was 0.1%, It has been shown to increase to up to 8% in those over 80 years old [6]. In a study by Karaçağlar et al., the average age of the patients was 70.4 years [7]. In our study, the mean age was 70.1±10.3 years, which is consistent with the literature. Physical activity plays an important role in reducing mortality and morbidity rates and in avoiding cardiac and vascular diseases. A study conducted in Africa showed that a sedentary lifestyle is a risk factor for AF. It was particularly emphasized that retirement is a risk factor for AF [8]. In our study, 48.8% were housewives, 41.7% were retirees, and 9.5% were currently employed. Although smoking and drinking are risk factors for AF [5], in our study, most patients with AF did not smoke or drink.

In our study, 77.4% of the patients took new-generation oral anticoagulants and 22.6% took warfarin. This is due to the fact that warfarin dose must be adjusted according to therapeutic indices, the INR must be monitored to be safe [6,9], and its use creates difficulties as it interacts with food and other drugs.Of patients using warfarin, less than 60% were within the therapeutic range, and patients were reluctant to take it due to its interactions with food, alcohol, and drugs [10,11]. The risk of intracranial bleeding is seven to 10 times higher in patients being treated with warfarin (INR 2.5-4.5), and the mortality rate in case of intracranial bleeding is 60% [12]. Under warfarin treatment, 59-67% of the patients were found to be at the targeted INR level [13]. Concerns about the side effects of warfarin, including bleeding, facilitated the transition to newer oral anticoagulants. Despite warfarin’s proven efficiency, there has been a search for new molecules that can be tracked easily and have no drug interactions, leading to the development of new oral anticoagulants [9]. If targeted INR values are not achieved despite training patients monitored via INR, then new oral anticoagulant treatments are recommended instead of warfarin [14].

Our study showed that the most preferred drug of the new oral coagulants was rivoraxaban 20 mg. In the ROCKET AF study, in patients with AF, rivoraxaban has been found to be as effective as warfarin in preventing systemic embolism and stroke [10]. The first approved oral anticoagulant, dabigatran, decreased the risk of systemic emboli in comparison to warfarin in the Randomised Evaluation of Long-term Anticoagulant Therapy (RE-LY) study [11]. According to the results of the ARISTOTLE (Apixaban for Reduction in Stroke and Other Thromboembolic Events in Atrial Fibrillation) study, apixaban is the third new-generation oral anticoagulant approved. When compared with warfarin, apixaban is more effective in preventing ischemic and hemorrhagic stroke primary endpoint for AF patients [12]. The higher use of new-generation oral anticoagulants compared to warfarin in our study is also supported by the literature. Studies show that these three new-generation oral anticoagulants used to prevent stroke and systemic endpoints of AF do not cause worse outcomes than warfarin. It has been shown that new-generation oral anticoagulants are not affected by age, gender, or body mass index. It has also been suggested that they will be the first choice for anticoagulant treatment unless there is a contraindication. New-generation oral anticoagulants also do not require adjustment of the drug dose​​​​​[15]. In our study, we found that more than half of the patients had been using new-generation oral anticoagulants for more than one year.

In long-term oral anticoagulant treatment, it is important to raise the awareness of patients about side effect profiles, interactions with other drugs, and applying to the emergency department [16]. In our study, we observed that 78% of the patients were informed by the doctor who prescribed the drug. However, most of the patients were unaware of the interactions of the oral anticoagulants they used, and how they would be affected if they forgot to take their pills or take too high a dose. Similarly, we observed that they did not have any information about the effects of the anticoagulant drugs they took, the time it took for the effects to appear, and when the effects would pass. This may be due to the low education level of the group comprising the study population and the advanced age group. Yaylacı et al. showed in their study that prescribing physicians did not have much information about the use of new-generation oral anticoagulants and warfarin and the side effects of anticoagulant drugs used for treatment [17]. In our study, 82% of the patients had no side effects from taking new-generation oral anticoagulants. No side effects were reported in 17% of patients taking warfarin. In a study indicating the importance of patients receiving information and education on warfarin use, when patients were properly trained, a reduction in side effects was been observed. Another study showed that patients' average information score rose to 8.1±1.2 from 4.7±2.8 after education related to warfarin and its side effects [17,18]. In a study conducted with warfarin, it was shown that the education given to the patient by the healthcare team has a positive contribution in informing the patient. This is in line with other studies [4,19,20,21]. In severe clinical conditions caused by warfarin, the new-generation oral anticoagulants can safely be used as an alternative in treatment [22].

## Conclusions

Guidelines updated by the American Heart Association specify that warfarin (proof level I-A), dabigatran (proof level I-B), rivaroxaban (proof level IIa-B), and apixaban (proof level I-A) are drugs of preference in AF treatment. In addition, European guidelines state that new-generation anticoagulants are superior to warfarin (proof level II-A). Informing the patient about the oral anticoagulants they take and their side effects will reduce hospitalization as well as contribute to a better quality of life and medical service use of AF patients. When patients start taking oral anticoagulants, information about the drug should be given and educational activities should be continued during the controls. The continuity of the education provided should be ensured. In particular, the awareness of patients about warfarin use should be increased. If the targeted INR cannot be achieved and complications arise, then the use of new-generation oral anticoagulants should be considered instead of warfarin therapy.
